# Implementation and applications of EMOD, an individual-based multi-disease modeling platform

**DOI:** 10.1093/femspd/fty059

**Published:** 2018-07-06

**Authors:** Anna Bershteyn, Jaline Gerardin, Daniel Bridenbecker, Christopher W Lorton, Jonathan Bloedow, Robert S Baker, Guillaume Chabot-Couture, Ye Chen, Thomas Fischle, Kurt Frey, Jillian S Gauld, Hao Hu, Amanda S Izzo, Daniel J Klein, Dejan Lukacevic, Kevin A McCarthy, Joel C Miller, Andre Lin Ouedraogo, T Alex Perkins, Jeffrey Steinkraus, Quirine A ten Bosch, Hung-Fu Ting, Svetlana Titova, Bradley G Wagner, Philip A Welkhoff, Edward A Wenger, Christian N Wiswell, for the Institute for Disease Modeling

**Affiliations:** 1 Institute for Disease Modeling, Bellevue, WA, USA; 2 Department of Biological Sciences, University of Notre Dame, Notre Dame, IN, USA; 3 Unit for Mathematical Modeling of Infectious Diseases, Institut Pasteur, Paris, France

**Keywords:** mathematical modeling, epidemiological modeling, software

## Abstract

Individual-based models provide modularity and structural flexibility necessary for modeling of infectious diseases at the within-host and population levels, but are challenging to implement. Levels of complexity can exceed the capacity and timescales for students and trainees in most academic institutions. Here we describe the process and advantages of a multi-disease framework approach developed with formal software support. The epidemiological modeling software, EMOD, has undergone a decade of software development. It is structured so that a majority of code is shared across disease modeling including malaria, HIV, tuberculosis, dengue, polio and typhoid. In additional to implementation efficiency, the sharing increases code usage and testing. The freely available codebase also includes hundreds of regression tests, scientific feature tests and component tests to help verify functionality and avoid inadvertent changes to functionality during future development. Here we describe the levels of detail, flexible configurability and modularity enabled by EMOD and the role of software development principles and processes in its development.

## Teaser

To address multi-scale questions in infectious disease transmission, a multi-disease modeling platform EMOD has been developed with modularity, code sharing across diseases, and formal software development and testing processes.

## INTRODUCTION

In 2007, Bill and Melinda Gates announced support for a new global commitment to malaria eradication (Bill Gates—Malaria Forum 2007). Numerous areas of specialization must converge to realize this goal, combining knowledge about human demographics, mobility, immunity, climate and mosquito breeding with current and novel interventions and implementation strategies. The call for eradication highlighted the need for an analytical tool to combine insights across these fields (WHO 2008). The same is true for other infectious diseases, which require interdisciplinary coordination to plan, implement and monitor programs to achieve disease control and elimination (Levin *et al.*^1997^). A computational platform was needed to bring together insights across these disciplines and estimate their impact on disease transmission.

One approach to bridging disciplines and scales of computational analysis is the use of individual-based models (IBMs), an approach that allows for great flexibility in structural assumptions, but that has only become computationally feasible in recent decades (Judson 1994). Though flexible, IBMs are more complex to create than traditional compartmental models (Railsback and Grimm 2011). Rather than the occupancy of states (for example, the number of infected individuals), they track individuals explicitly, allocating computer memory for each individual and propagating the individual's state and interactions through time. The software development requirements for IBMs pose a challenge, especially if a new framework needs to be developed from scratch.

Here, we summarize the decade-long development of a freely available individual-based disease modeling framework, EMOD (Bershteyn *et al.*^2012^; Eckhoff and Wenger 2016), and how it has expanded beyond malaria to include other infectious diseases that share a common framework. We discuss how multiple layers of detail become intertwined in the dynamics of transmission and the diverse group of professionals required to make this level of software complexity feasible and reliable.

EMOD has been developed over more than a decade by professional software developers, and so it has capabilities that would be very difficult to develop at an academic institution with shorter-duration PhD and post-doctoral trainee positions. By making the code and documentation openly available, the team hopes to enable infectious disease researchers, policy-makers, implementers and funders to access modeling software that is difficult to produce with the time and staffing more commonly available in academic and similar organizations.

## STRUCTURING IBMs FOR INFECTIOUS DISEASE DYNAMICS

IBMs, also known as agent-based models or micro-simulation models, use computer simulation to track interacting individuals and their environment to better understand disease transmission on a population level. Individuals are explicitly represented in computer memory from the time they are initialized (typically at birth) until the time they are removed from the population (typically at death), with the individual's attributes changing through internal processes and interactions. The models represent the history of each individual and participants in each interaction, while also allowing for population-level aggregate statistics to be computed. Such models are especially useful in fields such as ecology (Grimm and Railsback 2013), economics (Tesfatsion 2002), and infectious diseases (Garnett *et al.*^2011^)—areas in which autonomous individuals modify and are modified by others and their environment to give rise to population-level trends.

IBMs are particularly well-suited for creating infectious disease transmission models and studying intervention impact because they allow for attributes to be added to infections, individuals and their environments without exponentially increasing the total number of items (e.g. compartments) to be tracked. It is important to capture biological attributes of individuals and the pathogens they harbor, which inform how the individual's state propagates forward in time. At the same time, it is critical to capture the mechanisms by which diseases are transmitted, their environmental determinants, and the effect of interventions.

**Figure 1.**
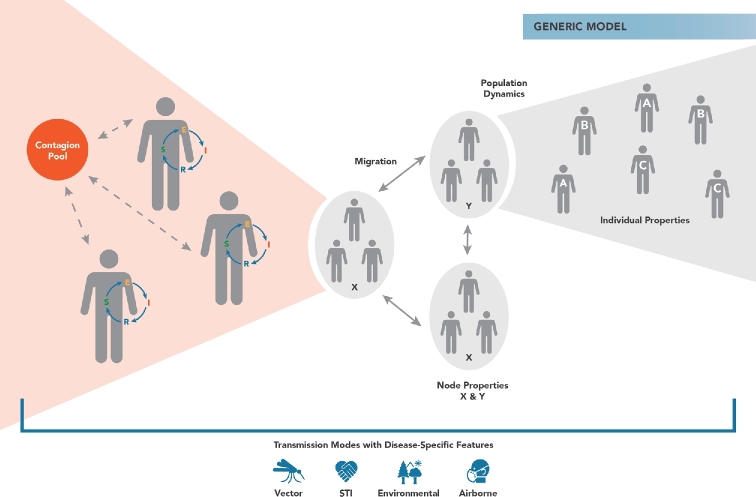
Diagram of the Generic transmission model in EMOD, in which the within-host disease process is represented as progression through the states of susceptible (S), Exposed (E), Infected (I) and Recovered (R). The dynamics of a compartmental SEIRS model can be produced when infected individuals shed infectiousness into a shared contagion pool (red circle) and susceptible individuals acquire from this pool. The Generic model allows for multiple contagion pools to be created based on geographic location (node) and modified based on the properties of the node. EMOD allows for individuals to have differential shedding and exposure according to demographic strata, including user-defined strata called individual properties. By representing SEIRS as internal states of the individual rather than compartments of the population, EMOD allows the transmission mode to be modified—for example, to a relationship network for sexual transmission—while the individual disease progression continues to follow the same SEIRS states.

The simplest within-host model available for EMOD is the Generic model, which simply reflects the susceptible-exposed-infected-recovered (and potential return to susceptible) model in epidemiology, often referred to as the SEIRS model (May and Anderson 1979; Keeling and Rohani 2011). Whereas a compartmental SEIRS model tracks the number of individuals in each of the states (susceptible, exposed, infected and recovered), an IBM implementation of SEIRS moves these states into the modeled individuals, as shown in Fig. 1. This gives the model flexibility to add demographic variables and dynamics, as well as flexibility determine how transmission might occur, while still preserving SEIRS as the internal disease states of the individual. To mimic a typical compartmental SEIRS model, transmission rates are simply the product of the number susceptible (S) and the number infected (I) in the population. The IBM equivalent of this calculation is for individuals in the infected population (those with internal state I) to deposit their contagion into a shared pool, and for susceptible individuals (those with state S) to acquire infectiousness from that pool. Without altering the internal SEIRS disease process, such a structure allows for the transmission mechanism to be switched, e.g. by adding an explicit contact network of remembered relationships between agents.

**Figure 2.**
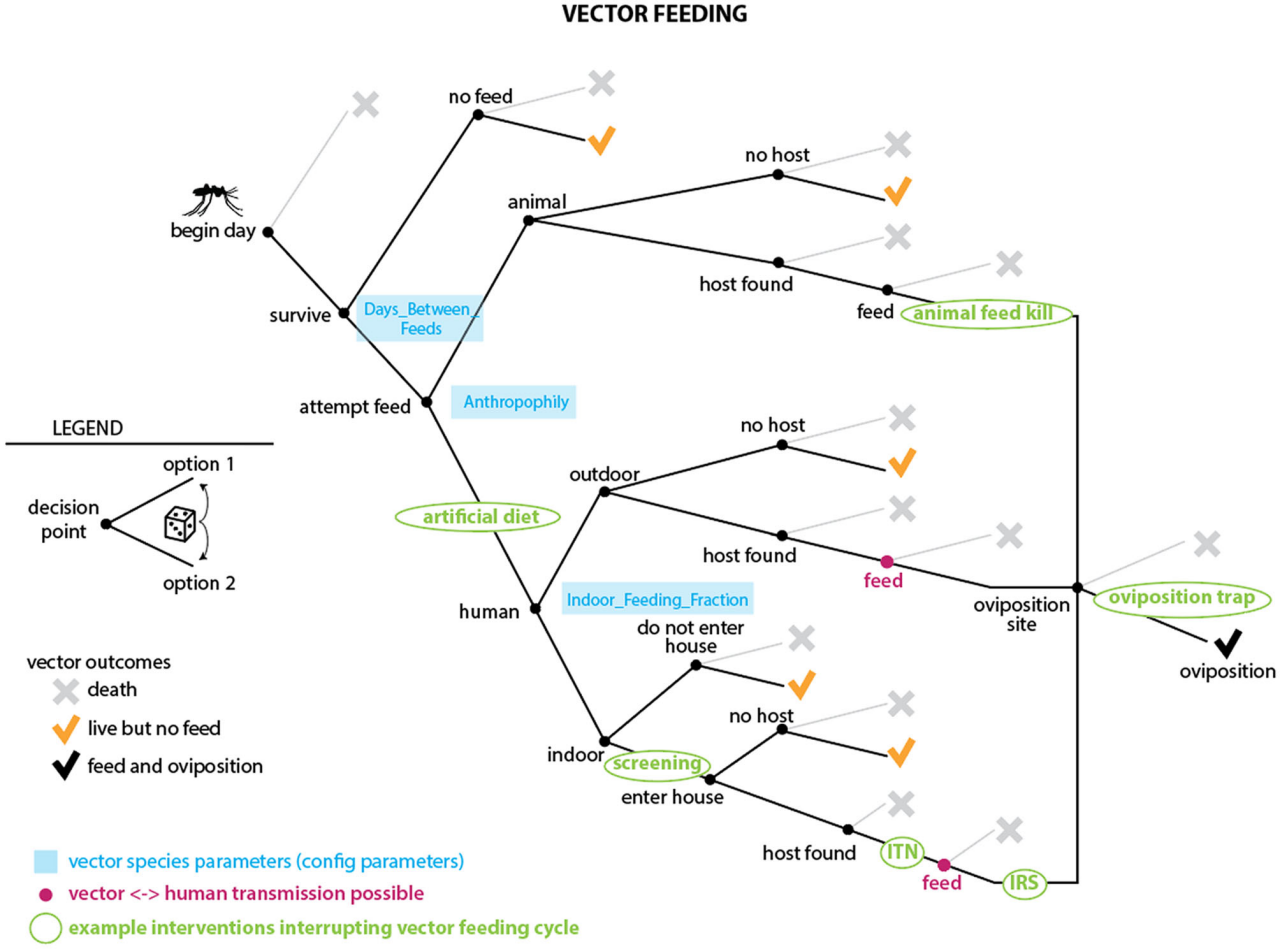
To illustrate how systems can have multiple points of interaction with interventions, this diagram shows a decision tree representing possible outcomes for an insect vector such as a mosquito in the EMOD vector model. This branching tree of conditional probabilities is used to determine the proportion of vectors that die, survive, feed and reproduce each day. Multiple simultaneous interventions can target various branches in the vector feeding tree, and the deterrent and toxic effects of multiple interventions can be represented simultaneously. For example, both indoor residual spraying (IRS) and insecticide-treated nets (ITN) can be applied against indoor host-seeking mosquitoes. IRS can discourage mosquitoes from entering the house and also kill mosquitoes before feeding. The fraction of mosquitoes that survive can be blocked by the ITN, which may also kill a subset of the blocked fraction. Those mosquitoes who survive the feeding attempt may be killed by IRS post-feed. Vector behavior parameters (blue) can be configured independently for each vector species being modeled, and the interventions (green) can be configured with multiple parameters such as the distributions of their blocking and killing abilities, duration of effect, duration of use and cost.

**Figure 3.**
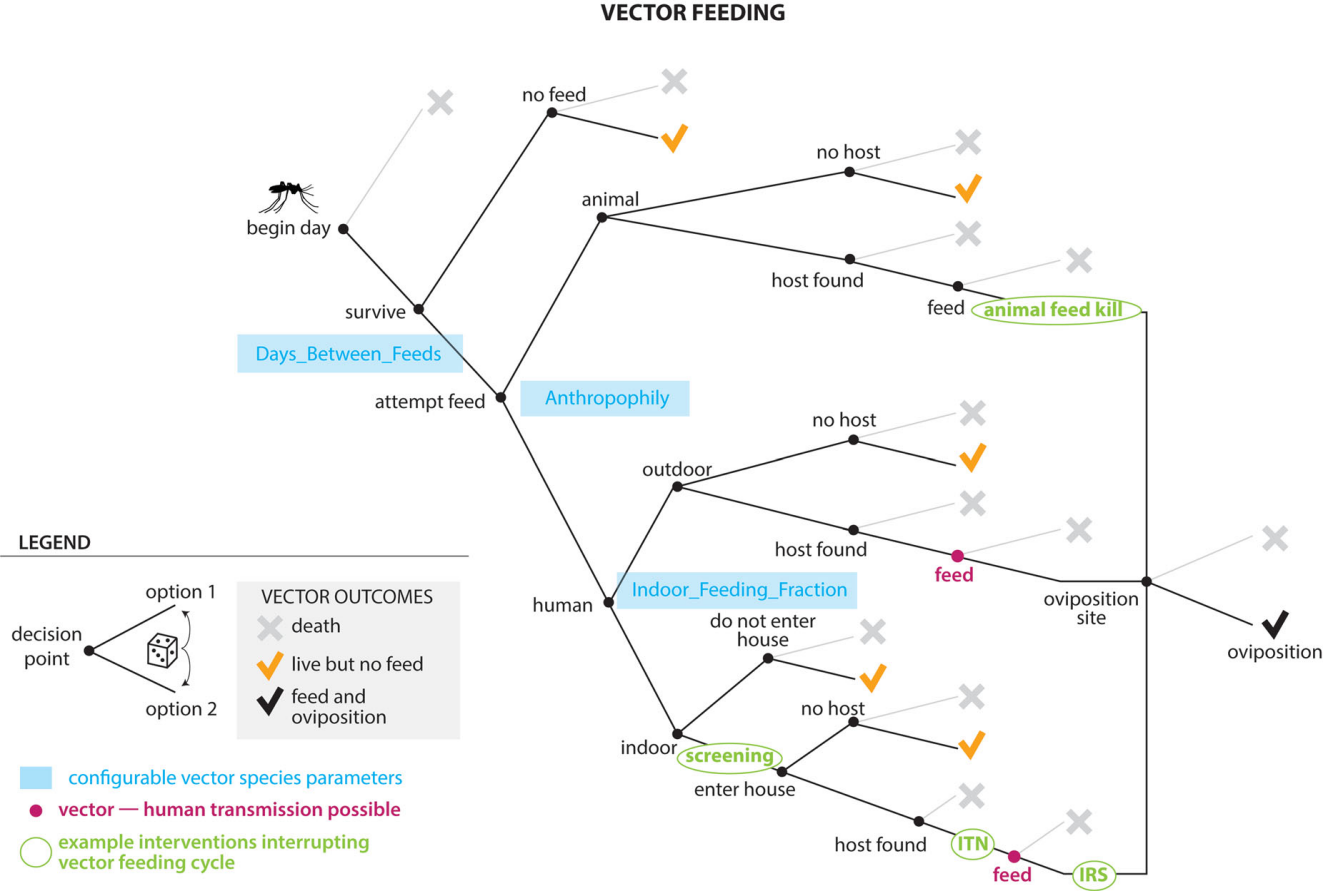
Network diagram describing how the components of the malaria model are organized into modules. Modularity ensures that specialists in a particular field, such as antimalarial drug pharmacokinetics or malaria infection and immunity, can add detail or develop new modules in their area of specialty while reducing the risk of inadvertently changing other components of the model. Even with modularity, software testing is required to formally show that a change was isolated to one feature of the model and did not inadvertently change other features. Similarly, it is necessary to check that a change to the common framework made by one disease specialist did not cause undesirable changes for other diseases supported by the common framework. For this reason, EMOD comes with (as of March 2018) over 600 regression tests and over 140 SFTs. The tests are run daily with the latest build of the EMOD codebase to automatically flag which model components have been modified.

More complex modeling of transmission modes can be necessary not just to capture transmission dynamics, but also to ensure that the impacts of interventions and their interactions can be accounted for. For example, for vector-borne diseases such as malaria, interventions can target multiple points in the vector's life cycle, as illustrated in Fig. 2, a diagram of the feeding cycle in the EMOD vector-borne disease model (Eckhoff 2011). The daily vector survival, feeding, vector-to-human disease transmission and egg-laying model is itself complex, but is just one module of the larger model illustrated in Fig. 3. Other modules include other parts of the vector life cycle (eggs, larvae and immature mosquitoes), human demographics, the within-human portion of the malaria life cycle, human health-seeking behaviors and health care delivery and human-focused interventions such as drugs and vaccines.

Because of their complexity, model components such those in Fig. 3 need to be designed with clear delineation and interfaces between modules so that model developers can make modifications to one component without disrupting other components. Modules also need to be configurable and swappable. For example, the adult vector model in Fig. 2 offers two options: the model can be configured to track all vectors (or a weighted sub-sample of vectors) as individual entities or vectors can be modeled compartmentally, tracking only categories of states and their occupancy. Using the individual model enables additional attributes such as migration preferences for habitat type and human habitation to be configured. While this level of detail can be useful for specific research questions (Gerardin *et al.*^2017^; Eckhoff *et al.*^2017^), it requires substantial computational resources. The compartmental model for vectors speeds up simulation time by tracking the number or proportion of vectors in each state. Modularity of code ensures that the different options for modeling vectors (Fig. 2) seamlessly plug in to the rest of the model (Fig. 3), making detail and configurability more tractable.

## STRUCTURE AND PURPOSE OF A MULTI-DISEASE MODELING PLATFORM

In infectious disease models, the mechanism of interaction between individuals is usually specific to the mode of disease transmission, an attribute shared by multiple diseases. For example, airborne diseases depend primarily on the rate at which infectious and susceptible individuals are co-located for transmission of particles in the air. Susceptibility, pathogenesis and infectiousness may be influenced by individual age, health and immune status, as well as environmental factors such as seasons and indoor air circulation. High-resolution environmental data sets are constructed to be inputs into EMOD simulations for detailed spatial models (Chabot-Couture, Nigmatulina and Eckhoff 2014). Vector-borne diseases depend on interactions of susceptible individuals with infected vectors, as well as infected individuals with susceptible vectors, in the respective life cycles of the pathogen. Environmental factors such as temperature and rainfall influence vector populations, whereas socio-demographic and immune factors influence human exposure and disease progression. Sexually transmitted diseases depend on person-to-person sexual interactions, which are structured into long-term relationships that form and dissolve according to individual attributes, preferences, living conditions and social norms.

It is also necessary at times to add more structural specificity pertaining to the disease being modeled, beyond the mode of transmission and its parameters. This is often the case for the internal processes that represent disease progression, and for disease-specific interventions such as diagnostics, treatments and vaccines that behave in disease-specific ways. For example, a generic model of a sexually transmitted infection (STI) may have simple disease states such as susceptible, exposed, infected and recovered (SEIR). However, modeling HIV requires more detailed representation of disease progression, such as of CD4 + T cell counts, their depletion during infection, reconstitution during antiretroviral treatment, impact on other infections such as tuberculosis and measurement for clinical decision-making. Adding a CD4 + T cell count property to all STI models would be unnecessary for modeling many other STIs, and thus should be applied only to HIV-specific models.

In addition to these transmission- and disease-specific considerations, there are major components of IBMs that are not specific to diseases or transmission modalities. For example, all IBMs require infrastructure for input of parameters, output of results, disease-independent demographics (initialization, births, non-disease deaths, migration and stratification by relevant biological and socio-demographic factors). Spatio-temporal patterns of health care access and delivery can have disease-specific components, but if made sufficiently configurable, much of this infrastructure can be shared across diseases. The disease-agnostic infrastructure of an IBM is often the most time-consuming and complex to create initially, but can be shared across disease models if the code is designed in a re-usable way, made available and maintained after the initial research applications are complete.

While the use of IBMs for infectious disease modeling is growing, re-usable platforms for modeling different diseases are uncommon. A recent review of IBMs for infectious disease transmission found nearly 700 published IBM studies, with increasing publication frequency over time, but little evidence of re-usable code architectures being applied across disease areas (Willem *et al.*^2017^). To address this problem, EMOD is architected to maximize sharing of code across disease areas. This not only conserves effort, but also increases utilization of the shared code and test cases, increases code coverage and opportunities to find bugs and provides features with applications across diseases, such as detailed demographics and care seeking patterns.

**Figure 4.**
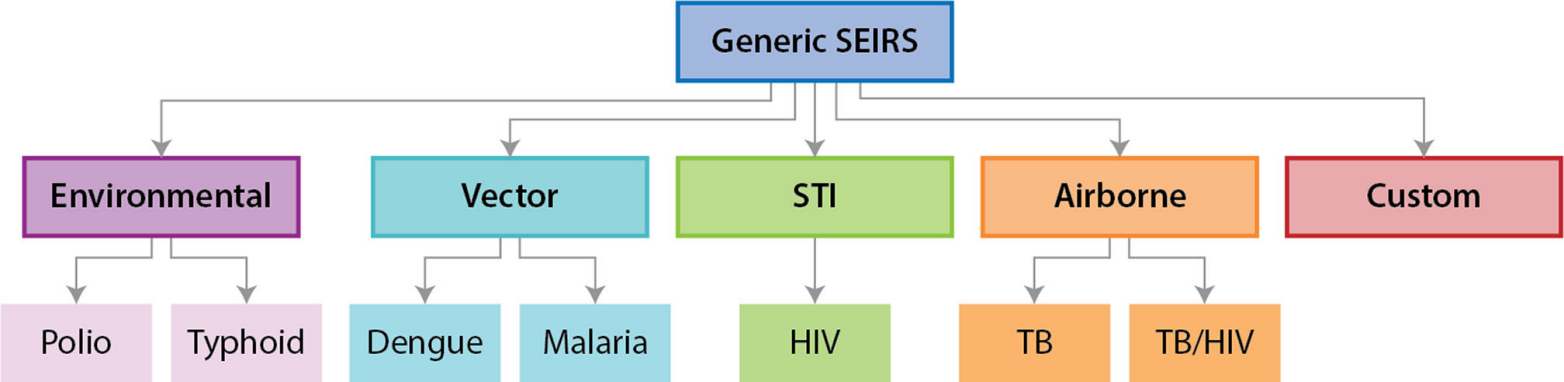
Inheritance structure of EMOD, illustrated for four disease types. Simulations can be run at each layer of specificity. In Generic SEIRS, individuals have generic disease states of susceptible, exposed, infected and recovered (SEIRS) and have properties such as date of birth, current age, sex, location, rates of fertility and mortality and socio-demographic strata, which are inherited by more specific disease models. Transmission mode models, such as airborne, vector or sexual transmission, retain the generic SEIRS disease states, but modify how individuals interact to give rise to transmission events. For example, sexual transmission adds types of sexual relationships and demographically-specific propensities to enter and break them. The HIV model inherits the sexual behavior logic, but over-writes the generic SEIRS disease and interventions with HIV-specific states and interventions.

Object-oriented programming (OOP) provides a facile means of sharing of generic code across disease areas, while still allowing for separation of specific code across disease types. Through inheritance, OOP classes for disease-specific objects such as individual humans, environments in geographic locations and pathogen infections can derive from generic classes. To span the generic functionality, transmission-mode-specific interactions and disease specificity, the individual human object in EMOD is architected with OOP using the inheritance structure shown in Fig. 4. At the generic level, individuals have properties such as date of birth, current age, sex, location and other socio-demographic categories. Individuals experience a risk of mortality due to causes other than the disease being modeled, and women experience age-specific fertility rates. Generic disease states include susceptible, exposed, infected and recovered. Generic interventions include diagnostics with a configurable sensitivity and specificity, and vaccines with a configurable rate of take and efficacy.

Each transmission type inherits the properties of the generic simulation, but adds features specific to the mode of transmission. For example, in the STI model, individuals have propensities to form and break sexual relationships and use general STI interventions such as condoms. The HIV disease-specific model extends the STI model by adding within-host attributes specific to HIV, such as CD4 + T cell counts and clinical stages of AIDS, as well as HIV-specific interventions such as antiretroviral therapy and male circumcision. The HIV model also over-writes some properties of the generic disease, replacing the susceptible/exposed/infected/recovered states with HIV-specific states such as acutely infected, chronically infected and symptomatic with AIDS. This structure has the advantage of efficiently sharing features that are applicable for all infectious diseases, and transmission-specific features for diseases that share a transmission mode.

The EMOD model provides a flexible framework to build simulations with different scales and different spatial resolutions so as to match the policy question of interest. For example, a polio model of Kano state (built upon the generic code base) simulated over 1322 nodes to represent the different clusters of populations across the state so as to capture the dynamics of the final chains of transmission, and to understand how long after the last case of the disease was detected could we be sure that the disease had been eliminated (McCarthy, Chabot-Couture and Shuaib 2016). In contrast, a polio model of 16 West African countries was also built using the EMOD software where each sub-national administrative unit (state or province) for each country was simulated as a single node. The resulting 210 nodes were used to study the propagation of vaccine infections following the outbreak response vaccination campaigns during the polio endgame using live poliovirus vaccine (McCarthy *et al.*^2017^).

Another advantage of the inheritance structure in EMOD is the ability to add a new disease with less effort, since much of the generic framework and transmission-mode-specific logic is already available. To make it easier to add new disease models, EMOD also supports a ‘custom’ disease class in which researchers supply a Python script to describe the disease process. EMOD is written in C^+^^+^ for computational speed, but Python is a more commonly used programming language in research and allows for rapid prototyping on top of the EMOD framework.

**Figure 5.**
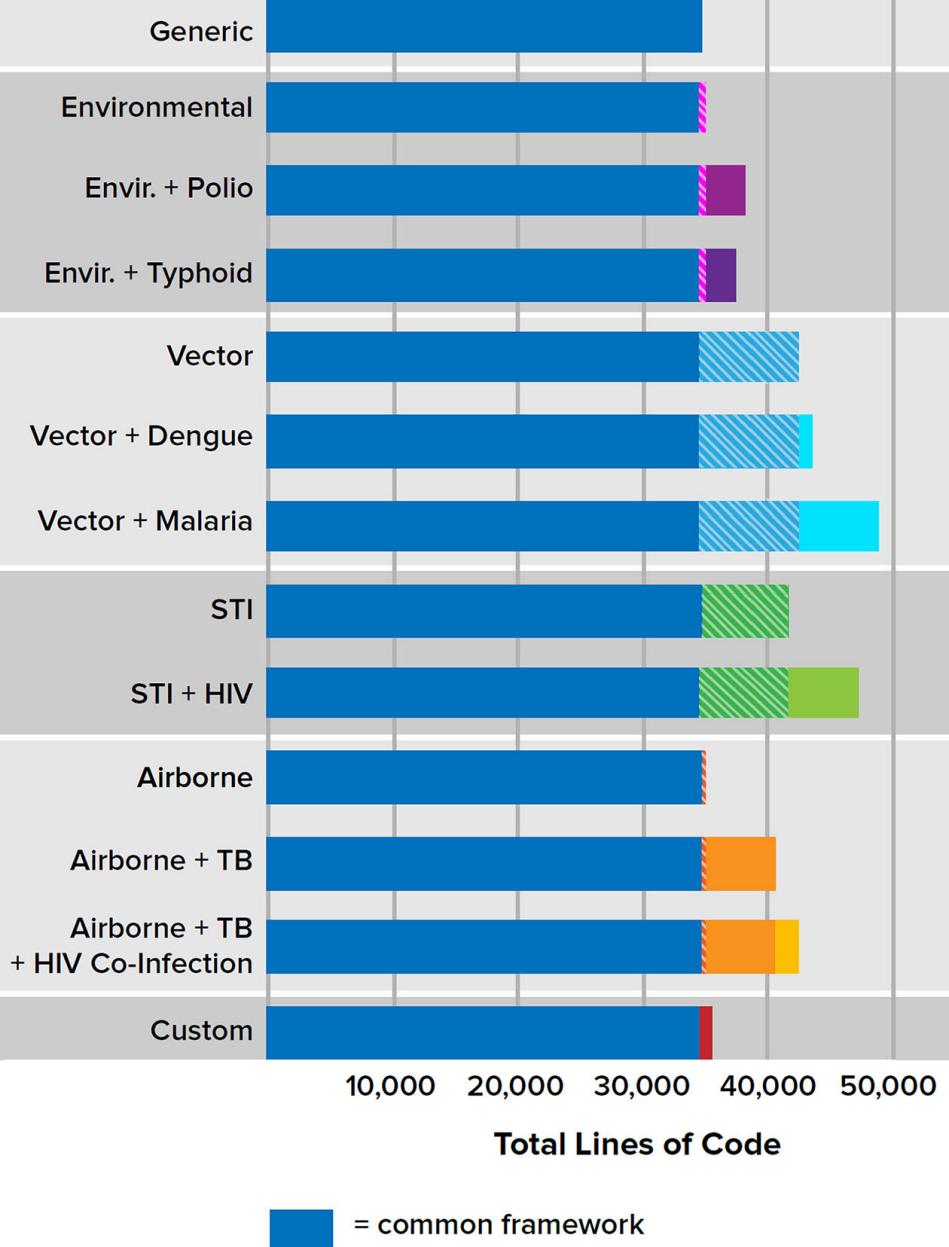
Number of lines of code available to different disease models, stratified by level of the inheritance hierarchy. Code was analyzed in February 2018 using Code surveyor by Construx Software, omitting lines that were blank or contained only comments. The common framework, sufficient to implement Generic SEIRS transmission and inherited by all other disease types, occupies 34 512 lines of code (dark blue bars). Cross-hatched areas indicate code that specifies transmission modality. Colored regions show disease specific code, which over-writes SEIRS with disease-specific states and interventions. As of this writing, no disease exceeds 50 000 lines of code. The vast majority of model code is devoted to the common framework, allowing for sharing of features and test cases across disease research.

To visualize the relative ease of adding new diseases to the EMOD framework, compare the number of lines of code devoted to the generic EMOD, transmission code and disease-specific code (Fig. 5). The common framework, which is inherited by all disease types and sufficient in itself to run the generic SEIRS model, occupies over 34 000 lines of code. The specific diseases are built on top of this common framework such that no specific disease model has required over 50 000 lines of code total. For example, to support vector-based research, about 8000 lines of code are added to the common framework code in order to model vectors—their lifecycle, transmission of the infection between vectors and humans and interventions that interrupt this transmission. The malaria model takes these combined 42 000 lines and adds another 6000 lines to model the specifics of malaria parasites, the development of partial immunity (Eckhoff 2012a,b) and other attributes that are not shared with other vector-borne diseases.

Because the EMOD source code is openly available, institutions are able to leverage the common framework to model diseases not currently included. For example, researchers at the University of Notre Dame have used the same vector model as a base for creating a model of dengue transmission. Because many core capabilities such as demographics and vector dynamics were already available in the generic vector model code, the dengue-specific model required only about 1000 additional lines of code. Sharing a majority of code between malaria and dengue not only reduces the workload of creating the models, but also increases code usage to further add confidence that the code is working correctly.

## SUPPORTING POLICY AND IMPLEMENTATION PLANNING THROUGH FLEXIBLE INTERVENTIONS

One of the most common reasons to modify disease transmission model code is to change structural assumptions about interventions, especially patterns of health-seeking behavior and health care delivery. For example, uncertainties about loss to follow-up from HIV care profoundly influence the estimated impact and cost-effectiveness of treatment programs (Bershteyn *et al.*^2015^). To constrain these uncertainties and make informed forecasts, models must to rapidly adapt to incorporate new evidence about the outcomes of those who appear to be lost to follow-up based on clinic records (Haas Andreas *et al.*^2018^; Holmes *et al.*^2018^), interventions to retain (Murray *et al.*^2017^) or re-engage lost patients (Bershteyn *et al.*^2017^; do Nascimento, Barker and Brodsky 2018), and the impact of other health system changes such as decentralization and differentiated care on patient retention (Bor *et al.*^2017^; Murray *et al.*^2017^). Making changes to health care patterns in the EMOD source code required re-compiling and re-testing the model, which limited the speed and frequency of updates to the health system model.

To address this problem, the ‘campaign’ framework in EMOD was developed to allow user input to flexibly configure the series of triggers, decisions, delays and targeting/filters that represent the process of providing interventions to individuals. The framework can also be used to deliver targeted seeding of new infections (required for initializing the epidemic) and adding or removing co-factors for transmission. For example, in the HIV model, the campaign framework is used to both create co-infections with other STIs that increase HIV susceptibility or infectiousness, and to deliver STI treatment.

**Figure 6.**
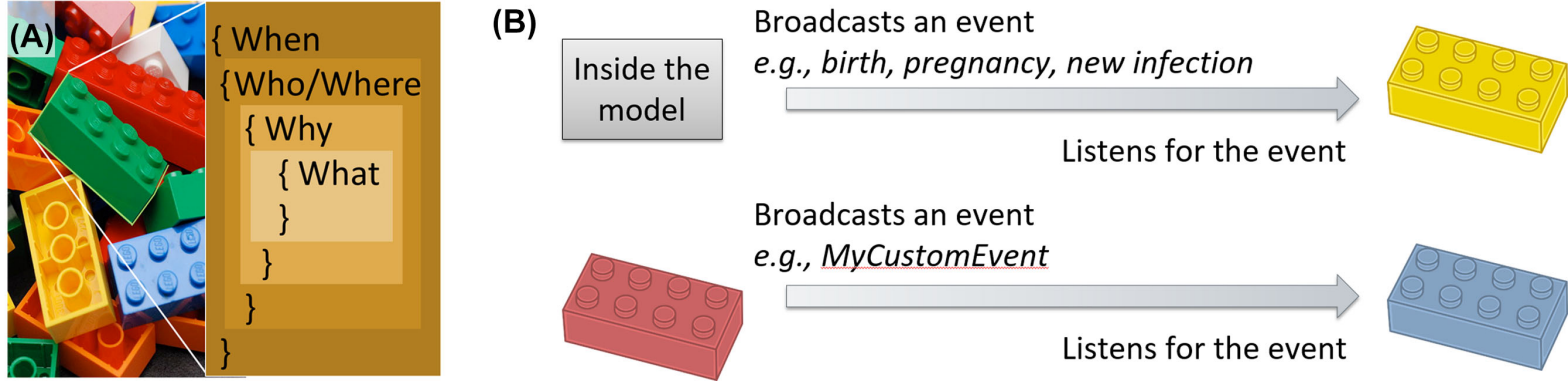
Conceptual illustration of how the EMOD campaign framework links together building blocks to enable a user-defined health care system and health-seeking behaviors. Individual building blocks specify the time that the intervention becomes available; the target population based on socio-demographic strata such as age, sex, location and user-defined characteristics such as risk group or accessibility; and the triggering condition for the intervention to be applied, if any. The building blocks are specified in JSON format read in by EMOD. The blocks are connected through broadcasts and triggers to build up series of events such as decisions, delays, diagnostics and interventions. The campaign framework can also be used to initialize new infections and to define the dynamics of co-factors for transmission, such as acquisition and treatment of STI co-infections that influence HIV transmissibility and susceptibility. Image credit for photograph in (**a**): ‘ Brand new bricks ’ by fdecomite, CC BY 2.0.

A user-defined ‘campaign’ file is formatted as JavaScript Object Notation (JSON) and consists of individual blocks, each specifying who, what, when, where and optionally why an intervention is to be applied, followed by the ‘what’ of the intervention itself (Fig. 6a). The ‘who’ includes both filtering on socio-demographic strata and the ability to select a random proportion of the chosen stratum. The ‘what’ includes the intervention and the parameters to configure it, e.g. the time-course of efficacy and take rate of a vaccine, or the specificity and sensitivity of a diagnostic. The ‘when’ component may specify an absolute time in the simulation to model an historical event or a planned future intervention. The ‘where’ component may restrict the intervention to specific geographic locations in the model. The ‘what’ component is not necessarily an intervention; it can also simply cause the individual to broadcast a user-defined name of the event.

This broadcast ability underlies the power of the campaign framework: it enables users to chain together series of custom-defined steps. To do so, the user can also add a ‘why’ component of the intervention block, which listens for a specified broadcast and only delivers an event when triggered by that broadcast. When the broadcast of one campaign block matches the trigger of another, it links the two events together in a chain. As illustrated in Fig. 6b, the broadcast can originate from dynamic events within the model, such as births, deaths, migration, pregnancies, new infections or formation of new relationships. Alternatively, the trigger can be a user-specified broadcast coming from another campaign block. Thus, without modifying the source code, users can build up complex logical flows of decisions, delays, diagnostics and interventions, all with configurable timing, probabilities and targeting to sub-populations.

**Figure 7.**
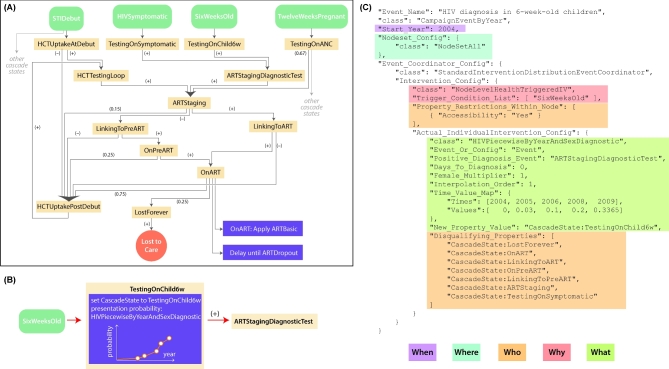
Example of a typical user-defined set of health-seeking and health care patterns. These components, sequences of flow between them, and parameters governing their effects are provided by the user and fully configurable. Panel (**a**) shows a schematic of the HIV interventions related to HIV testing and treatment, which are provided as input into the EMOD model for HIV in South Africa. It is composed of many individual building blocks, diagramed here as purple boxes in (**b**) and encoded as JSON objects as in (**c**). The sub-section of (a) that is expanded in (b) and (c) is HIV testing of children. The building blocks specify the time that the intervention becomes available (Start_Year); the target population based on socio-demographic strata such as age, sex, location and user-defined characteristics such as risk group or accessibility (‘Accessibility’: ‘Yes’); and the triggering condition for the intervention to be applied, if any. The triggering condition can be one arising from the internal dynamics of the model (such as becoming six weeks of age, as in the example above, ‘SixWeeksOld’) or can be a user-defined event arising from another building block (such as the output of the example above, ‘ARTStagingDiagnostic Test,’ which will trigger the next event to happen as shown by the red arrow in (b)). Building blocks may act as probabilistic decisions, delays, diagnostics and interventions. Some building blocks allow for additional user input, for example, the probabilistic decision of whether to be tested for HIV in (b) and (c) allows user to input a time-series for the probability of HIV testing. EMOD will linearly interpolate the time-series to assign a probability at each simulation time step, as shown in the orange graph in (b).

As an example of a complex care system that can be fully user-defined, Fig. 7 shows a typical ‘cascade of care’ used in the EMOD HIV model, and zooms into one component (infant HIV testing) to show how health seeking patterns are user-specified. The baseline model and resulting epidemic and treatment patterns have been systematically compared to country-specific data and other model estimates (Eaton *et al.*^2012^, ^2014^, ^2015^), and the ability to flexibly target sub-populations has been used to explore strategies for efficient resource allocation and interrupting onward transmission (Klein, Eckhoff and Bershteyn 2015; Bershteyn, Klein and Eckhoff 2016). The triggerable campaign framework has also been used to configure reactive case investigation in a spatial malaria model, where treatment of an index case in a household broadcasts the signal for case investigation only to neighboring households (Gerardin *et al.*^2017^).

The flexible campaign framework allows for rapid response to policy and implementation questions by adding *de novo* structures to health-seeking and health delivery models without the need to modify model code. However, other structural changes to model assumptions, such as addition of new features or new disease types, do require code modifications. In the following section, we describe the software processes and quality assurance measures used validate and maintain EMOD.

## SOFTWARE DEVELOPMENT PROCESSES IN DISEASE MODELING

The Institute for Disease Modeling has an innovative organizational structure. Working alongside teams of disease researchers are professional software developers, testers and documentation specialists, most having years of experience at software companies such as Microsoft. This unique combination of specialists brings software discipline to the process of model development, including the individual-based infectious disease modeling framework, EMOD. As a shared resource among disease specialists and sub-specialists, the software team enables re-use and coordination of software components across disease applications. For example, the triggerable care cascade framework was originally developed for HIV, but has since been applied in other disease areas such as malaria and tuberculosis; detailed vaccine framework was originally developed for polio and malaria modeling, but has been leveraged for HIV modeling. The source code for EMOD is available online at https://github.com/InstituteForDiseaseModeling/EMOD.

To develop new features or disease applications using EMOD, researchers from the team and collaborating institutions may choose to contribute code to the model, or to provide detailed specifications to a software developer who will implement the new feature or change. Code changes are subject to code review by a designated maintainer of the code repository. After a feature is added, the researcher who requested it is tasked with designing a test case to ensure that the feature works as specified from a scientific perspective. In addition, professional software testers perform further testing to assess technical adherence to the specification, as well as other factors such as informative error messages and handling of corner cases.

The size and collaborative development of the EMOD codebase necessitates a more formal level of testing, including automation of testing. Software developers generally perform some degree of testing in the course of model development, ensuring that new feature is behaving as expected. The simplest form of testing consists of ‘trying out’ the new feature with a set of EMOD input files and inspecting the output files for the anticipated result. However, it is also necessary to ensure that the changes did not inadvertently alter some other aspect of the model—potentially in a different disease area than the one for which the feature was intended.

To address this issue, the feature-specific tests (model input and the expected output) that are created in the course of development are stored over time and aggregated into a set of test cases known as regression tests. EMOD currently includes over 600 tests producing over 1200 output files, available online at https://github.com/InstituteforDiseaseModeling/EMOD/tree/master/Regression. When a new feature is added, it is recommended to run the entire batch of regression tests in order to check whether the code changes have altered the output of any previous test case. At IDM, the tests are automatically run nightly with the latest version of the EMOD code. After a nightly run, the files are compared against the previous day's output and changed files are flagged for further investigation.

The regression test system alerts the team to any test for which the output files are non-identical to the previous day's output, regardless of whether the changes to the output files are substantive. It is not uncommon for these differences to be ‘false alarms.’ In the case of Monte Carlo agent-based models, a change to the number or sequence of calls to a random number generator can alter the output of a given simulation run, but would not change the scientific behavior of the model, e.g. average result of multiple stochastic replicates.

To enable model testing when random number streams have changed, and more generally, to provide a more thorough comparison of the model's behavior relative to its scientific specification, the team has developed more intensive tests termed Scientific Feature Tests (SFTs), of which over 140 are currently available at https://github.com/InstituteforDiseaseModeling/EMOD/tree/master/Regression (a separate folder of SFTs is available for each disease type). An example of an SFT is checking whether random draws from a specified probability distribution really do follow the expected distribution, e.g. by repeatedly sampling from the distribution and ensuring that it is statistically unlikely to differ from the expectation. SFTs can sometimes be thought of as ‘black-box testing’ because they test the model's behavior based on its outputs, regardless of the internal implementation.

In contrast to the ‘black box testing’ approach of SFTs, a ‘white box testing’ approach is also taken to examine the internal workings of the software itself, including implementation-specific aspects of the software that are not user-facing. Such tests are typically referred to as ‘component tests’ or ‘unit tests’ and sometimes require separate code, known as test harnesses, to instantiate and examine individual components of the model in isolation. Available component tests for EMOD can be found at https://github.com/InstituteforDiseaseModeling/EMOD/tree/master/componentTests. Component tests allow for verification of model functionality when subtle problems with a feature might not be evident from the output of the overall epidemic simulation. For example, such a test might generate a population of individuals with unique identifiers, and then check whether the correct numbers of unique identifiers were created in memory. Other examples include verifying whether diagnostics appropriately exhibit the configured specificity and sensitivity, or whether sexual relationships interact appropriately with migration.

The development team also includes software documentation professionals. Technical documentation is available online at www.idmod.org/documentation and training materials are in development and have been piloted in university courses, on-site trainings and trainings held at collaborating institutions. In addition, the EMOD executable can generate a schema, or list of all parameters available in the version of the software being run. The schema includes parameter names, data types, defaults, ranges and short descriptions. A frontier for EMOD is learning the most effective way to support new users with a variety of goals, timelines and experience levels.

The configurability of EMOD makes the model suitable for multiple purposes: it can be used to triangulate baseline data for estimates of disease trends, to forecast impact of interventions with detailed patterns of uptake and to connect with economic models to address resource allocation questions. The model can output both individual-level event histories and population-level trends, allowing for examination of epidemic drivers and testing of ideas for efficient interruption of disease transmission.

However, EMOD also presents challenges and limitations that make it less suitable for some purposes. Configurability comes at the cost of ease-of-use, especially for the health care and health-seeking patterns that can be re-structured by users with innumerable possible configurations, rather than being selected from a fixed list of options. The effort required to become and remain familiar with the model and how to configure parameters is significant, often taking weeks or months depending on the task. This makes EMOD less suitable for producing results very rapidly (days to weeks) in new settings where the model has not already been configured and fit to baseline epidemic or endemic data.

The data and effort required to parameterize and fit EMOD to specific settings also make it more suitable for settings in which detailed data are available, particularly data stratified by population categories such as age groups and geographic regions. In principle, most forms of model detail can be disabled or configured to mirror a simple assumption (for example, birth rates can be made proportional to population size and mortality rates can be age, sex and time invariant). However, computational and ease-of-use limitations make EMOD an unlikely choice for simulations that could be conducted with simpler compartmental models. Instead, EMOD is typically applied in data-rich settings.

Considering the effort required to fit EMOD to a new setting, it is unlikely that EMOD will be applied systematically to all settings affected by diseases, as has been done with other modeling software in order to produce estimates in all countries of the world with a systematic and harmonized approach. To date, applications of EMOD have favored detailed examination of settings in which rich and emerging data provide insights into epidemic drivers and intervention impact, and heuristics from such detailed modeling can then be applied elsewhere, but usually without re-fitting the model to all settings where the heuristic may apply.

Efficient, automated approaches to model calibration are an area of active development at IDM. Challenges of calibrating EMOD include the large number of model parameters that can be modified through calibration, the computation time required to run the model (typically on the scale of minutes to tens of minutes, depending on how the model is configured) which limits the number of times the model can be run as part of calibration, and the stochastic nature of the model, which adds further uncertainty to model outputs that requires more model runs to be quantified. Improved efficiency and ease of calibration would allow EMOD to be applied to more settings and more easily updated as data become available.

## CONCLUSIONS

Dynamic modeling of infectious diseases using IBMs brings together a diverse set of disciplines at different scales of detail, from within-host biological processes to between-host interactions to population-level patterns. The interaction of these components brings about a level of complexity that requires software discipline and formal testing and benefits greatly from sharing of code across disease applications to maximize code usage for efficiency and debugging. We have described the experience of developing EMOD, a multi-disease modeling framework that uses a modular approach to define interactions between model components, and OOP to maximize sharing of features across disease applications.

With support from Bill and Melinda Gates through the Global Good Fund, EMOD is available free of charge, and source code is openly available. The model can be compiled to run on recent versions of Windows and multiple Linux distributions, and can be run on a laptop or, particularly for large spatial simulations, on a supercomputer or cloud computing environment. EMOD is a unique resource to the disease modeling community, both for those who wish to work with currently available disease models, and for those seeking to build upon the common framework to model different infectious diseases, including those that may emerge in the future.
